# A Three-Stage Inter-Channel Calibration Approach for Passive Radar on Moving Platforms Exploiting the Minimum Variance Power Spectrum

**DOI:** 10.3390/s21010069

**Published:** 2020-12-24

**Authors:** Philipp Wojaczek, Diego Cristallini, Daniel W. O’Hagan, Fabiola Colone, Giovanni Paolo Blasone, Pierfrancesco Lombardo

**Affiliations:** 1 Fraunhofer Institute of High-Frequency Physics and Radar Techniques FHR, Fraunhoferstrasse 20, 53343 Wachtberg, Germany; diego.cristallini@fhr.fraunhofer.de (D.C.); daniel.ohagan@fhr.fraunhofer.de (D.W.O.); 2 Department of Information Engineering, Electronics and Telecommunications DIET, Sapienza University of Rome, Via Eudossiana, 18, 00184 Rome, Italy; fabiola.colone@uniroma1.it (F.C.); giovannipaolo.blasone@uniroma1.it (G.P.B.); pierfrancesco.lombardo@uniroma1.it (P.L.)

**Keywords:** PCL, moving platforms, passive radar, calibration, DPCA, clutter suppression, minimum variance power spectrum

## Abstract

Research in passive radar has moved its focus towards passive radar on moving platforms in recent years with the purpose of moving target indication and ground imaging via synthetic aperture radar. This is also fostered by the progress in hardware miniaturization, which alleviates the installation of the required hardware on moving platforms. Terrestrial transmitters, commonly known as illuminators of opportunity in the passive radar community, usually emit the signals in the Very High Frequency (VHF) or Ultra High Frequency (UHF) band. Due to the long wavelengths of the VHF/UHF band, there are constraints on the size of the used antenna elements, and therefore, the number of antenna elements to be employed is limited, especially as the platform carrying the passive radar system is intended to be small, potentially even an unmanned aerial vehicle. In order to detect moving targets hidden by Doppler shifted clutter returns, one common approach is to suppress the clutter returns by applying clutter suppression techniques that rely on spatial and temporal degrees of freedom, such as Displaced Phase Center Antenna (DPCA) or Space-Time Adaptive Processing. It has been shown that the DPCA approach is a meaningful technique to suppress the clutter if two antenna elements are employed. However, if the employed receiving channels are not carefully calibrated, the clutter suppression is shown to be not effective. Here, we suggest a three-stage calibration technique in order to perform the calibration of two receiving channels, which involves the exploitation of the direct signal, a data-adaptive amplitude calibration, and finally, a data-adaptive calibration of phase mismatches between both receiving channels by the estimation of the Minimum Variance Power Spectrum of the clutter. The validity of the proposed approach is shown with simulated data and demonstrated on real data from a fast ground moving platform, showing improved clutter cancellation capabilities.

## 1. Introduction

The concept of Passive Coherent Location (PCL) or passive radar has gained renewed research interest in recent years [1,2,3], due to the advantages that PCL offers. As a passive radar system does not emit any electromagnetic signal, neither a dedicated Transmitter (TX) for target illumination needs to be maintained nor an approval for spectrum allocation is necessary. Instead, it only receives and exploits electromagnetic waves emitted by so-called Illuminators of Opportunity (IO). Therefore, a passive radar is relatively inexpensive and low cost, making it especially interesting for civilian applications. Another great advantage, especially for military users, is the fact that the passive radar has a low probability of being detected, as it does not emit any signals.

Usually broadcast TXs, as well as communication transmitters are used as IO, among others, e.g., FM radio (Frequency Modulated radio), DVB-T (Digital-Video-Broadcast- Terrestrial) [4], or GSM (Global System for Mobile Communications) [5]. Typically, DVB-T signals are emitted with a high Equivalent Isotropically Radiated Power (EIRP) to cover large areas. Furthermore, as it is a digitally modulated waveform, there is the possibility to decode and remodulate the transmitted signal in order to have a clean reference signal [6]. This, together with the fact that the bandwidth of approximately 8 MHz provides a fair equivalent monostatic range resolution of approximately 20 m, makes DVB-T one of the most used illuminators for passive radar. As the passive radar system does not need to maintain a transmitter (thus being low-power) and due to the progress in hardware, which leads to miniaturization and more powerful systems, it becomes possible to mount the passive radar receivers on moving platforms, for example small boats or even unmanned aerial vehicles [7].

The first results of airborne passive radar dedicated to Moving Target Indication (MTI) were presented in [8,9,10,11]. In [12], the application of the CLEAN filter and its limits for ground clutter removal were presented. (Unfortunately, the authors were not able to find the spelling of the acronym “CLEAN”. The CLEAN filter was first introduced in 1974 by Högbom in [13] for image filtering in radio astronomy, however without providing the spelling of CLEAN. The authors believe that the name “CLEAN” of the filter refers to the fact that the images of the sky appeared “dirty” and therefore needed to be “cleaned”, as also outlined in ([13], p. 420).) The publications in [14,15] focused on reference signal estimation and clutter analysis. A critical point to be considered for the performance of a passive radar for target detection is the knowledge about the position of the TX and the accuracy of the TX position. This was analyzed in [16,17].

The main drawback of a PCL system is that it is impossible to control the transmitted waveform, as it was designed for communication purposes, which makes radar signal processing more challenging. For instance, the DVB-T standard describes the use of so-called “pilots”, which are used for synchronization purposes and channel estimation [18]. Due to their deterministic and periodic character, pilots can hinder target detection as they generate ambiguities and sidelobes, which may overlap target echoes in the resulting range-Doppler maps. In addition, also the non-deterministic and time-varying data carriers might prevent target detection. Specifically, in [19,20], it was analyzed in detail how the deterministic and non-deterministic data prevent clutter suppression and limit the target detection both in the endo-clutter and the exo-clutter region. It was suggested there to apply the Reciprocal Filter (RpF) for range-compression. The RpF is shown not only to remove ambiguities arising from the periodic signal structure, but it also ensures a time-invariant range compression response, which is a required condition for applying an efficient clutter suppression technique, e.g., DPCA. Performing in this way is similar to a pulse-Doppler radar. In [19], it was further suggested to use the DPCA technique to remove the Doppler modulated clutter returns. For the technique DPCA, at least two displaced antenna elements are used, which are mounted along-track in the side-looking condition [21,22,23]. Here, the front/back antenna elements are labeled as Leading Antenna (LA)/Trailing Antenna (TA). As the receiver platform is moving, the TA will occupy the spatial position of the LA a time step later, provided that the receiver platform motion is linear without changes in flight attitude. This is commonly known as the DPCA condition. Consequently, under the assumption of stationary clutter, the TA will see the same phase shift of the clutter returns as the LA, while a moving target experiences a phase shift due to its own radial motion relative to the transmitter and receiver, which is different in both receiving channels. Therefore, after compensation of the time delay due to the spatial displacement between LA and TA, which is known as co-registration, the clutter returns can be removed via simple subtraction of the range-compressed data, while the target echo is preserved.

Due to environmental influences and physical limitations, e.g., wind speed leading to changes in flight attitude or non-constant flight velocity, the fulfillment of the DPCA condition is not guaranteed. To re-establish the DPCA condition in digital post-processing, the technique “flexible DPCA” (or short flex-DPCA) was suggested in [19].

However, DPCA is also very sensitive to non-idealities and errors in the characteristics of the RF receiving channel [24]. Therefore, a careful calibration between both receiving channels has to be applied to the received signals in order to achieve a complete clutter suppression.

The topic of receiving channel calibration for DPCA was addressed first in [25], by applying the simple approach of exploiting the direct signal interference for calibration. Although this simple approach has been shown to be effective, still a clutter residual remains after clutter suppression. Especially for non-uniform antenna patterns, the effectiveness of this technique can be very limited. In fact, a calibration based on the sole exploitation of the direct signal does not take advantage of the multipath signals (i.e., of the clutter signal) impinging the receiver antenna from multiple directions. This approach was further extended to calibrate the phase variation of the antenna pattern for each Doppler-bin independently [26]. It operates data-adaptively in the angle-Doppler domain, namely on the estimated Minimum Variance Power Spectrum (MVPS) [23,24]. The technique has been shown to be effective in clutter suppression by applying it against real data from a slow-moving maritime platform. However, in [26], an amplitude calibration was not performed.

In [27,28,29,30], calibration techniques were suggested in order to cope with geometry induced clutter non-stationarity for active bistatic radar.

In [31,32], a two-stage strategy operating data adaptively in the range-Doppler domain on specific regions was suggested. The strategy aims at first removing the Direct Signal Interference (DSI) from the illuminator and second at calibrating on the clutter returns to maximize the clutter suppression in a DPCA clutter filtering stage. Results were presented for simulated data and for real data evaluation from a ground moving platform.

In this paper, we present a new joint calibration of the amplitude and phase values for each Doppler-bin. This is based on the use of the principal eigenvalues in the range-Doppler domain for amplitude calibration, followed by a correction strategy approach for phase calibration operating in the angle-Doppler domain, that is by estimating the MVPS of the received data. We provide a simulated and experimental demonstration of the effectiveness of the proposed approach.

The outline of this paper is as follows: Section 2 gives an overview of MTI and introduces the DPCA technique. In Section 2, we define our signal model under the consideration of channel errors and extend the signal model for the spatial displacement compensation technique, i.e., flex-DPCA. In Section 3, we analyze the effects of calibration errors and the flex-DPCA technique on the MVPS analytically and in simulations. Section 4 introduces the calibration steps required for successful clutter suppression. The calibration algorithms, introduced in Section 4, are applied on real data in Section 5. Finally, in Section 6, our conclusions are drawn.

The notation in the paper is as follows: variables written in bold font in lower/upper case letters define vectors/matrices. The superscript “H” denotes the Hermitian operation.

## 2. Moving Target Indication and DPCA

MTI is one of the key operations performed by a radar mounted on a moving platform. It describes the capability of detecting moving targets against an interfering clutter background. The detection of moving targets is performed by exploiting the difference between target and clutter radial velocities and the consequent different Doppler frequencies [23]. Fast moving targets, here defined as targets with the Doppler frequency greater than the clutter Doppler frequency, can be detected by exploiting a one-channel receiver only. However, the Doppler frequency of a slow moving target, i.e., a target with radial velocity towards the receiver and transmitter smaller than the maximal radial velocity component of the clutter, will be inside of the clutter Doppler spectrum and potentially be hidden by the clutter background. For the detection of slow moving targets, multi-channel array processing is necessary in order to have enough degrees of freedom for clutter removal by creating a two-dimensional clutter filter in the angle-Doppler domain [23]. The angle-Doppler domain can be estimated, e.g., using MVPS estimation (also known as Capon super-resolution estimation) [24], which is described in detail in Section 3.

Two general multi-channel techniques exist, which enable and facilitate target detection against the clutter, namely DPCA and Space-Time Adaptive Processing (STAP) [23,33]. While STAP is very effective against homogeneous clutter due to the existence of a sufficient number of secondary data, it is not well suited for the removal of heterogeneous clutter. However, using DPCA, it is in principle possible, to achieve optimum clutter suppression in heterogeneous scenarios [34]. As the clutter returns are usually heterogeneous, we focus here on the DPCA technique.

Usually, two antenna elements N=2 mounted along-track are used: one LA and one TA. As outlined before, after compensation of the spatial displacement between the phase centers of LA and TA, the clutter can be suppressed via simple subtraction of the range compressed returns. In ideal conditions, the clutter would be removed up to the level of thermal noise.

Key requirements for DPCA are the fulfillment of the DPCA condition and calibrated antenna elements and hardware chains. This is usually difficult to fulfill in a real environment. In the following, a number of successive steps will be outlined that need to be performed to successfully implement DPCA for passive radar on moving platforms.

### Signal Model Including Channel Errors

Under far-field conditions, the signal back scattered from one single point-like scatterer and impinging from Angle of Arrival (AoA) α on LA and TA can be written as:

(1) $$\begin{array}{cc} s^{\left(\mathrm{LA}\right)}\left(t\right)= & s_{\mathrm{Tx}}\left(t\right)\exp\left(j2\pi \frac{v_{\mathrm{Rx}}}{\lambda }\cos\left(\alpha \right)\;t\right) \end{array}$$

(2) $$\begin{array}{cc} s^{\left(\mathrm{TA}\right)}\left(t\right)= & s_{\mathrm{Tx}}\left(t\right)\exp\left(j2\pi \frac{v_{\mathrm{Rx}}}{\lambda }\cos\left(\alpha \right)\;t\right)\cdot \exp\left(-j2\pi \frac{d}{\lambda }\cos\left(\alpha \right)\right) \end{array}$$

where $s_{\mathrm{Tx}}\left(t\right)$ refers to the transmitted signal in the time domain and $t\in [0,\ldots ,T_{\mathrm{Acq}}]$, $T_{\mathrm{Acq}}$ defining the duration of signal acquisition. λ, *d*, and $v_{\mathrm{Rx}}$ describe the wavelength of the center frequency $f_{C}$ of the transmitted signal, the antenna displacement, and the receiver’s velocity, which is here assumed to be linear and constant during signal acquisition.

In the following, and without loss of generality, we account for channel imbalances in the signal model of the TA, thus assuming the signal at the LA to be ideal. (2) is then updated as follows:

(3) $$\begin{array}{c} \begin{array}{cc} s^{\left(\mathrm{TA}\right)}\left(t\right)= & A\left(\alpha \right)s_{\mathrm{Tx}}\left(t\right)\exp\left(j2\pi \frac{v_{\mathrm{Rx}}}{\lambda }\cos\left(\alpha \right)\right)\cdot \exp\left(-j2\pi \frac{d}{\lambda }\cos\left(\alpha \right)\right)\cdot \exp\left(j\xi \left(\alpha \right)\right) \end{array} \end{array}$$

where A(α) and ξ(α) describe amplitude and phase imbalances dependent on the AoA α. Here, they are assumed to occur only in the antenna pattern. Furthermore, we assume a stationary scatterer, i.e., Internal Clutter Motion (ICM) is neglected here. Variations in the time delay between both channels are negligible here, as the preprocessing of the down-converted signals involves a synchronization on the same Orthogonal Frequency-Division Multiplexing (OFDM) symbol in both antennas, which is in the resolution of $f_{S}/2$ with $f_{S}$ defining the sampling frequency; see [25].

Analyzing (3), one notices three exponential terms. The first one describes the Doppler shift of the point like scatterer due to the receiver’s motion relative to the scatterer. The second exponential term describes the phase shift that is experienced in between the signal from the LA and the TA due to the antenna’s displacement. Finally, the third one describes the influence of the antenna phase errors.

Here, we range-compress the received signal by resorting to a batch-wise strategy, by using the RpF. This allows us to remove the signal’s information content [19] in order to achieve an equalized response of the output of the range-compression stage in frequency domain.

According to the aforementioned batch-wise range processing, after demodulation and sampling with sampling frequency $f_{S}$, the signals in (1) and (3) can be written in a discrete form as:

(4) $$\begin{array}{cc} s^{\left(\mathrm{LA}\right)}\left[l\right]= & \sum_{m}s_{m}\left[l-mL\right]\exp\left(j2\pi \frac{v_{\mathrm{Rx}}}{\lambda }\cos\left(\alpha \right)\;mT\right) \end{array}$$

(5) $$\begin{array}{c} \begin{array}{cc} s^{\left(\mathrm{TA}\right)}\left[l\right]=A & \left(\alpha \right)\sum_{m}s_{m}\left[l-mL\right]\exp\left(j2\pi \frac{v_{\mathrm{Rx}}}{\lambda }\cos\left(\alpha \right)\;mT\right)\\ \cdot & \exp\left(-j2\pi \frac{d}{\lambda }\cos\alpha \right)\exp\left(j\xi \left(\alpha \right)\right) \end{array} \end{array}$$

where $l=\lfloor f_{S}t\rfloor$ is the sampling index, m=[0,…,M−1] refers to batch *m* in the coherent processing interval with *M* batches, and $L_{S}=f_{S}T_{B}$ describes the number of samples in one batch of duration $T_{B}$. $T_{B}$ is assumed to be equal to the duration of one complete DVB-T symbol $T_{S}$. Due to the batch processing, an equivalent pulse-Doppler radar with Pulse Repetition Frequency (PRF) or Pulse Repetition Interval (PRI) of $\mathrm{PRI}=\frac{1}{\mathrm{PRF}}=T_{S}=T_{B}=T_{U}+T_{G}$ is created, where $T_{G}$ is the length of the guard interval.

After reciprocal filtering in the range compression stage using the reconstructed transmitted signal $\hat{s}\left(t\right)$ and by assuming an error-free reconstruction of $\hat{s}\left(t\right)$, the information content is removed, and the output of the range compression stage becomes [19]:

(6) $$\begin{array}{c} \begin{array}{cc} r^{\left(\mathrm{LA}\right)}\left[l,m\right]= & \sum_{m}\delta \left[l-mL_{S}\right]\cdot \exp\left(j2\pi \frac{v_{\mathrm{Rx}}}{\lambda }\cos\left(\alpha \right)\;mT\right) \end{array} \end{array}$$

(7) $$\begin{array}{c} \begin{array}{cc} r^{\left(\mathrm{TA}\right)}\left[l,m\right]= & A\left(\alpha \right)\sum_{m}\delta \left[l-mL_{S}\right]\cdot \exp\left(j2\pi \frac{v_{\mathrm{Rx}}}{\lambda }\cos\left(\alpha \right)\;mT\right)\\ \; & \cdot \exp\left(-j2\pi \frac{d}{\lambda }\cos\left(\alpha \right)\right)\cdot \exp\left(j\xi \left(\alpha \right)\right) \end{array} \end{array}$$

δ[l] refers to the Kronecker delta function, which is clearly independent of a DVB-T symbol’s content.

The output after the DPCA clutter suppression stage becomes:

(8) $$\begin{array}{c} r^{\left(\mathrm{DPCA}\right)}\left[l,m\right]=r^{\left(\mathrm{LA}\right)}\left[l,m-\gamma \right]-r^{\left(\mathrm{TA}\right)}\left[l,m\right] \end{array}$$

where the index γ refers to the motion compensation via the time delay of the data of the leading antenna.

A crucial requirement for (8) to be effective is the fulfillment of the DPCA condition:

(9) $$\begin{array}{c} v_{\mathrm{Rx}}=\frac{d}{T_{D}}=\frac{d}{KT_{S}},\;K\in N \end{array}$$

$T_{D}=KT_{S}$ is the required DPCA time delay between the LA and the TA, where *K* is an integer value.

In a real environment, the constraint in (9) might pose too stringent requirements to the system, thus resulting in (8) being ineffective. This means leaving a residual un-subtracted contribution after (8) even in the ideal case of perfectly stationary clutter. The so-called flex-DPCA technique provides the possibility to re-establish the DPCA condition and to compensate for the time delay; see [19]. It is based on the estimation of the receiver’s velocity using an Inertial Measurement Unit (IMU) on board the receiver. The application involves an integer time shift $T_{q}$ in the time domain, followed by a linear phase law correction $2\pi f_{D}\Delta T$ to be applied to each bistatic Doppler frequency $f_{D}$.

The calculation of $T_{q}$ and ΔT is as follows:

(10) $$\begin{array}{c} \begin{array}{cc} T_{q} & =\left\lfloor \frac{d}{T_{S}v_{\mathrm{Rx}}}\right\rfloor T_{S}\\ \Delta T & =T_{D}-T_{q},\;\Delta T\in \left[0,\ldots ,T_{S}\right] \end{array} \end{array}$$

Using $\gamma =T_{q}/T_{S}$, the motion compensated signal model in (6) is then:

(11) $$\begin{array}{c} \begin{array}{cc} r^{\left(\mathrm{LA}\right)}\left[l,m-\gamma \right]= & L_{S}\delta \left[l,m-\gamma \right]\cdot \exp\left(j2\pi \frac{v_{\mathrm{Rx}}}{\lambda }\cos\left(\alpha \right)\cdot \left(m-\gamma \right)T_{S}\right) \end{array} \end{array}$$

Using (11) in (8):

(12) $$\begin{array}{c} \begin{array}{cc} r^{\left(\mathrm{DPCA}\right)}\left[l,m\right]= & L_{S}\delta \left[l-\left(m-\gamma \right)L_{S}\right]\exp\left(j2\pi \frac{v_{\mathrm{Rx}}}{\lambda }\cos\left(\alpha \right)\;mT_{S}\right)-\\ \; & L_{S}A\left(\alpha \right)\delta \left[l-mL_{S}\right]\exp\left(j2\pi \frac{v_{\mathrm{Rx}}}{\lambda }\cos\left(\alpha \right)mT_{S}\right)\exp\left(j\xi \left(\alpha \right)\right)\\ = & L_{S}\delta \left[l-mL_{S}\right]\exp\left(j2\pi \frac{v_{\mathrm{Rx}}}{\lambda }\cos\left(\alpha \right)\;mT_{S}\right)\cdot \left(1-A(\alpha )\exp(j\xi (\alpha ))\right) \end{array} \end{array}$$

where the identity of $\delta \left[l-mL_{S}\right]=\delta \left[l-\left(m-\gamma \right)L_{S}\right]$ was used. We would like to highlight the last term: 1−A(α)exp(jξ(α)). This term represents the missing calibration, which is responsible for the residual after clutter subtraction, i.e., the application of DPCA. In the case of perfectly calibrated antenna elements (and provided the stationarity of clutter), A(α)=1 and ξ(α)=0 would hold, which would lead to an ideal clutter subtraction.

## 3. Effects of Channel Errors and Flex-DPCA on the MVPS Distribution

The MVPS displays the clutter in the angle-Doppler domain. This gives the possibility to analyze the clutter returns for each Doppler-bin related to the AoA, in order to study the effect of flex-DPCA and the influence of antenna pattern calibration errors.

We simulated a receiver with two antenna elements displaced with d=λ/2 mounted along-track. It was assumed that the elements have a uniform pattern and receive in the angular domain of $\Theta =[-85^{\circ },\ldots ,85^{\circ }]$ relative to the boresight. The assumption of antennas with a uniform pattern is valid, as typically, omnidirectional antenna elements are employed in order to achieve an omnidirectional coverage in PCL. The DPCA delay time $T_{D}$ was set deliberately to an integer multiple K=9 of the duration of one batch (DVB-T symbol, respectively), such that its velocity $v_{\mathrm{Rx}}$ amounts to (from (9)): $v_{\mathrm{Rx}}=\frac{d}{T_{D}}=\frac{d}{KT_{S}}=24.8016$ m/s. The MVPS $P_{\mathrm{MV}}$ can be estimated using [23,24]:

(13) $$\begin{array}{cc} P_{\mathrm{MV}}\left(f_{D},\bar{\theta }\right) & =\frac{1}{s^{H}\left(f_{D},\bar{\theta }\right)R^{-1}s\left(f_{D},\bar{\theta }\right)} \end{array}$$

where $s(f_{D},\bar{\theta })$ is a space-time steering vector for bistatic Doppler $f_{D}$ and the normalized angle of arrival $\bar{\theta }=\frac{d}{\lambda }\sin\theta$. R defines the estimated (clutter + noise) covariance matrix.

A comment on the estimation of the covariance matrix is in order: as was mentioned before, we did not consider the use of STAP, which involves covariance matrix estimation, for clutter suppression due to the non-heterogeneity of the clutter. However, for the estimation of the MVPS, it is also required to estimate a covariance matrix. The difference is in the goal of the estimation: the covariance matrix for STAP is required to estimate a two-dimensional filter, where the clutter in the training cells should follow the same clutter statistic as the cell under test, which is a requirement that can be difficult to fulfill. In the case of the MVPS, the covariance matrix is not used to design a filter, but instead to estimate the clutter in the two-dimensional plane. Therefore, the clutter non-heterogeneity can be neglected in the estimation of the MVPS.

**Figure 1:**
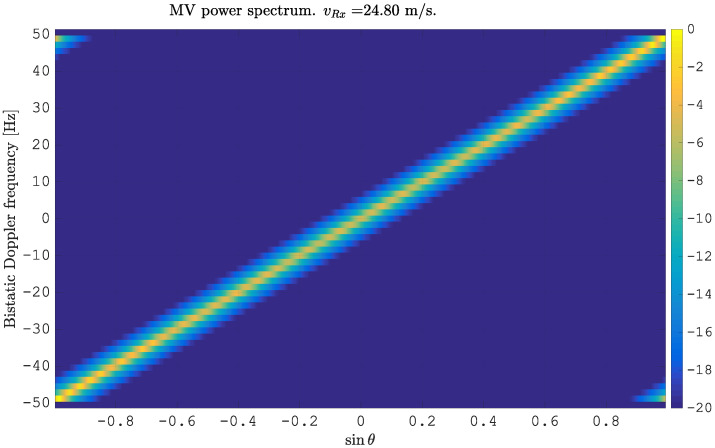
Minimum Variance (VM) power spectrum estimated from simulated clutter for N=2 spatially displaced antenna elements.

**Figure 2:**
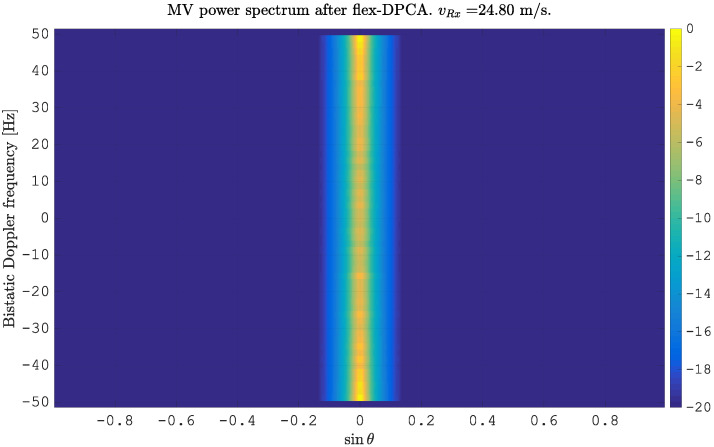
Minimum variance power spectrum estimated from simulated clutter for N=2 spatially displaced antenna elements after motion compensation using flexible Displaced Phase Center Antenna (flex-DPCA).

The estimated MVPSs are shown in Figure 1 and Figure 2. Figure 1 shows the MVPS before the flex-DPCA operation. As expected, the clutter spectrum is aligned along a diagonal in the angle-Doppler domain with slope $\beta =\frac{v_{\mathrm{Rx}}}{d\cdot \mathrm{PRF}}$. The clutter spectrum shows a uniform pattern, as uniform elements were simulated.

Compensating for the motion using a time delay according to (10) and re-estimating the MVPS lead to a vertical alignment of the clutter spectrum at $\sin\theta =0,\;\forall f_{D}$, as shown in Figure 2.

This is because the compensation of the motion is equivalent to the removal of the spatial displacement between the channels, or in other words: the phase centers of both antennas overlap spatially. Therefore, it is also not possible anymore to estimate the direction of arrival for each $f_{D}$.

**Figure 3:**
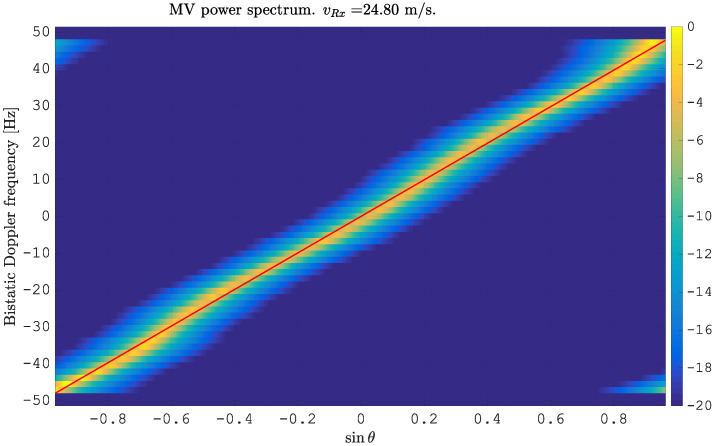
Minimum variance power spectrum estimated from simulated clutter for N=2 spatially displaced antenna elements with antenna pattern errors in amplitude and phase. The red line indicates the ideal slope of the clutter.

**Figure 4:**
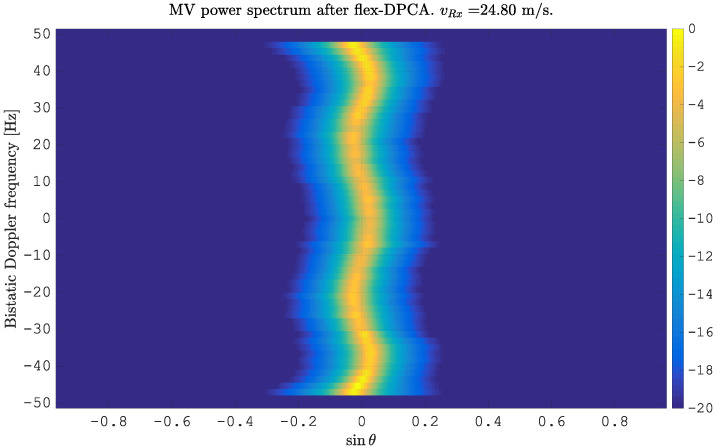
Minimum variance power spectrum estimated from simulated clutter after flex-DPCA. Due to antenna pattern errors, the maximum values show a severe deviation from the center.

In (3), the signal model for the TA including channel imbalances is introduced. According to this model, a simulation can be performed as well, in order to analyze the effects of channel imbalances on the clutter spectrum in the angle-Doppler domain. We extend the simulation presented before and implement amplitude and phase imbalances using a Covariance Matrix Taper (CMT) t as described in [24]. It is a vector of the form:

(14) $$\begin{array}{c} t=\left[A\left(\alpha_{1}\right)\exp\left(j\varphi \left(\alpha_{1}\right)\right),\ldots ,A\left(\alpha_{N_{\alpha }}\right)\exp\left(j\varphi \left(\alpha_{N_{\alpha }}\right)\right)\right] \end{array}$$

where $N_{\alpha }$ defines the number of simulated angles of arrival. (14) follows the form of antenna pattern errors defined in (3). The amplitude errors are randomly generated from the uniform distribution U{0.9,…,1.1} with arbitrarily chosen limits. The phase errors are simulated to follow a sinusoidal function with a frequency of $f_{\sin}=3$ Hz and an amplitude of $\varphi_{\mathrm{Amp}}=5^{\circ }$. In Figure 3, we report the simulated clutter in the angle-Doppler domain. The red line indicates the ideal position of the clutter without antenna pattern errors. One can clearly detect the deviation of the clutter spectrum from the red line, due to the changing width of clutter bins in the angular domain and due to the non-linear behavior of the clutter spectrum. The application of flex-DPCA projects all bins towards sinθ=0; however, the bins are not symmetrically centered around sinθ=0. Instead, they show a serious deviation of the maximum value from the center. This is shown in the following Figure 4. Obviously, an ideal clutter subtraction is not possible. The outcome of this is in accordance with (12): despite the identity of $\delta \left[l-mL_{S}\right]=\delta \left[l-\left(m-\gamma \right)L_{S}\right]$, which has been used in (12), the output of this DPCA stage will not become zero, due to the angle dependent channel imbalances in amplitude A(α) and phase ξ(α). The simple motion compensation is obviously not enough for removing the clutter completely. However, it is a crucial requirement and a necessary first step to be done for clutter subtraction.

## 4. Three Stage Calibration Approach

Obviously, in a real environment, the clutter spectrum will not be aligned totally at $\bar{\theta }=0$ after the application of flex-DPCA, due to non-perfect knowledge of the receiver’s velocity, non-collinear motion of the phase centers of both antenna elements, non-linear motion of the receiver (e.g., due to acceleration), errors or imbalances in the antenna pattern, receiving chain, etc. However, based on the above-mentioned conclusions (1) and (2), it is possible to exploit the MVPS for digital calibration with the goal to achieve a robust and data adaptive clutter suppression. Specifically, we propose a three stage data adaptive calibration. The first stage is coarse calibration and resembles the equalization of the direct signal interference from the TX received on the two channels. The second and third stages define a fine calibration, which is based on MVPS and accounts for amplitude and phase effects, respectively.

### 4.1. Digital Calibration on the Direct Signal

First, the antenna elements and receiving hardware can be calibrated digitally using the direct signal, which appears in a range-Doppler map in the range cell $r_{\mathrm{Tx}}$ and at Doppler frequency $f_{D_{\mathrm{Tx}}}=\frac{v_{\mathrm{Rx}}}{\lambda }\sin\theta_{\mathrm{Tx}}$.

The phase $\xi (\alpha_{\mathrm{Tx}})$ and the amplitude $A(\alpha_{\mathrm{Tx}})$ necessary for calibration can be calculated with:

(15) $$\begin{array}{c} \begin{array}{cc} \xi (\alpha_{\mathrm{Tx}}) & =\arg\left\{Z^{\left(\mathrm{TA}\right)}\left(f_{D_{\mathrm{Tx}}},r_{\mathrm{Tx}}\right)Z^{\left(\mathrm{LA}\right)}{\left(f_{D_{\mathrm{Tx}}},r_{\mathrm{Tx}}\right)}^{*}\right\}\\ A(\alpha_{\mathrm{Tx}}) & =|Z^{\left(\mathrm{LA}\right)}\left(f_{D_{\mathrm{Tx}}},r_{\mathrm{Tx}}\right)/Z^{\left(\mathrm{TA}\right)}\left(f_{D_{\mathrm{Tx}}},r_{\mathrm{Tx}}\right)| \end{array} \end{array}$$

where $Z^{\left(\mathrm{LA}\right)}$ and $Z^{\left(\mathrm{TA}\right)}$ define the complex range-Doppler data of the leading and the trailing antenna element. The data of the TA are then calibrated with:

(16) $$\begin{array}{c} {\hat{Z}}^{\left(\mathrm{TA}\right)}=A\left(\alpha_{\mathrm{Tx}}\right)Z^{\left(\mathrm{TA}\right)}\exp\left(j\xi \left(\alpha_{\mathrm{Tx}}\right)\right) \end{array}$$

In the following, this technique is referred to as Calibration on Direct Signal (CDS).

Following the theoretical analysis of [25], CDS has been proven to be a viable inter-channel calibration approach. However, this is only valid if the direct signal impinges the receiving array in the main-lobe [31].

### 4.2. Amplitude Calibration

An important point to be considered in the calibration process refers to the amplitude patterns of the respective antenna elements, which should be calibrated before calibrating the corresponding phases. The amplitude needs to be calibrated for each Doppler-bin or, equivalently, for each AoA. We note that this calibration is a difference from the CDS calibration based on the direct signal.

The calibration for the amplitude is angle and Doppler dependent and based on the estimation of a clutter covariance matrix $R_{C}$ and its eigenvalue decomposition, as defined in:

(17) $$\begin{array}{c} \begin{array}{cc} R_{C}\left(\alpha \right) & =\frac{1}{K}\sum_{k}^{K}Z\left(\alpha ,k\right)Z{\left(\alpha ,k\right)}^{H}\\ Z(\alpha ,k) & =\left[\begin{array}{c} Z^{\left(\mathrm{LA}\right)}\left(\alpha ,k\right)\\ {\hat{Z}}^{\left(\mathrm{TA}\right)}\left(\alpha ,k\right) \end{array}\right]\\ A(\alpha ) & =\left|\frac{e_{11}}{e_{12}}\right| \end{array} \end{array}$$

where k=1,…,K defines the index of the range cells taken into account for the estimation of $R_{C}$. The variables $e_{11}$ and $e_{12}$ define the components of the eigenvector $e_{1}$, which refers to the greatest eigenvalue $\lambda_{1}$ of the covariance matrix $R_{C}$. The amplitude calibration factor A(α) is explicitly shown as a function of the AoA α, but it can be expressed equivalently as a function of Doppler frequency $f_{D}$.

We observe here that the range cells considered for covariance matrix estimation need to be chosen carefully. Since the DSI is one strong component in the data, if it would be considered for covariance matrix estimation, then the resulting eigenvector components in (17) would reflect the DSI instead of clutter, and consequently, the DPCA stage would not cancel the clutter; see for this also the statistical analysis of clutter and DSI in [35]. Therefore, the first range cells with strong clutter components should be excluded from the estimation.

### 4.3. Phase Calibration

The next most crucial point to be considered is the calibration of the phase for each angle of arrival. As the antenna pattern is not uniform in the angle domain, an angle-dependent phase calibration needs to be applied. The calibration is based on an observation made in Section 3. There, it was stated that after motion compensation, the clutter spectrum will be aligned vertically at $\bar{\theta }=0\;\forall \;f_{D}$ in the angle-Doppler domain. However, channel imbalances lead to deviations of the peak of the clutter spectrum from the vertical axis. That means the clutter’s centroid of each Doppler-bin (but for the Doppler-bin where the DSI appears) potentially deviates from $\bar{\theta }=0$. The MVPS is a suitable representation domain for compensating this deviation: the angular deviation ${\bar{\theta }}_{e}\left(f_{D}\right)$ of the clutter centroid of each Doppler-bin $f_{D}$ can be estimated in the angle-Doppler domain $P_{\mathrm{MV}}\left(f_{D},\bar{\theta }\right)$. This can be done by searching for the maximum in each Doppler-bin of the angle-Doppler domain. The estimated values can then be applied as calibration factors on the range-Doppler map ${\hat{Z}}^{\left(\mathrm{TA}\right)}$ of the TA for each Doppler-bin for all range cells $k\in [1,\ldots ,N_{R}]$:

(18) $$\begin{array}{c} \begin{array}{cc} {\tilde{Z}}^{\left(\mathrm{TA}\right)}\left(f_{D},k\right)= & \exp\left(j2\pi d/\lambda {\bar{\theta }}_{e}\left(f_{D}\right)\right){\hat{Z}}^{\left(\mathrm{TA}\right)}\left(f_{D},k\right),\\ & k\in [1,\ldots ,N_{R}] \end{array} \end{array}$$

**Figure 5:**
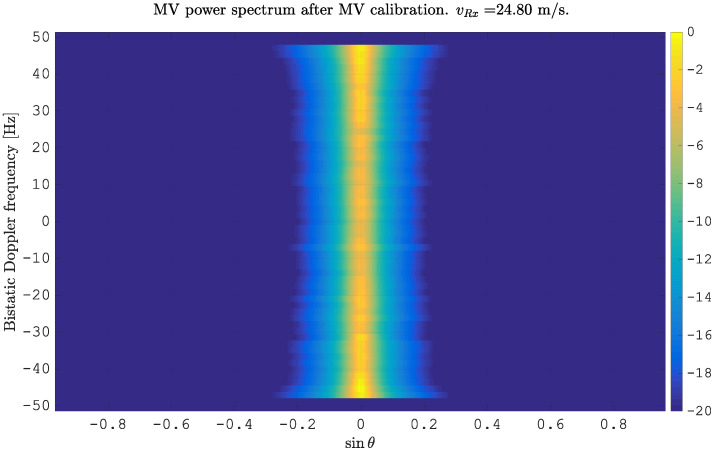
Minimum variance power spectrum estimated from simulated clutter after flex-DPCA and calibration using MVPS.

We applied this approach on the simulated MVPS shown in Figure 4 and present the result of this calibration in Figure 5. Clearly, the clutter spectrum is now aligned at $\bar{\theta }=0$ and can be canceled with DPCA.

**Figure 6:**
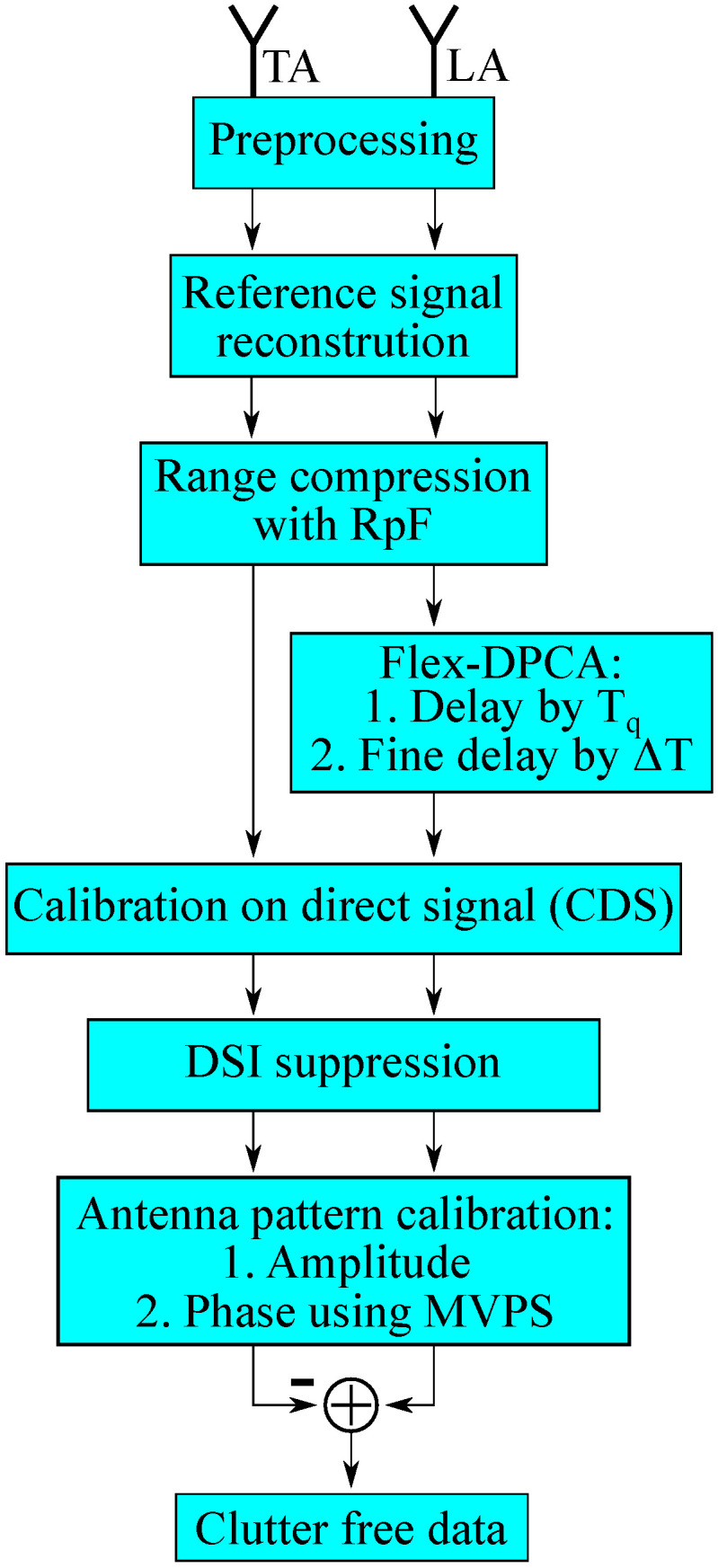
Flowchart of the processing. Preprocessing includes down-conversion, sampling, and synchronization on the direct signal. LA, Leading Antenna; TA, Trailing Antenna; DSI, Direct Signal Interference; MVPS, Minimum Variance Power Spectrum.

In order to support the understanding of the processing and keeping track of the involved calibration steps, we provide in Figure 6 a flowchart of the processing. After preprocessing, which involves down-conversion and sampling of the received signal and synchronization on the direct signal, the reference signal is reconstructed and used for range compression with the RpF. Afterwards, flex-DPCA is applied on the LA by applying the time delay in the time domain and the frequency domain. Typically in passive radar, the DSI is canceled in order to improve the signal-to-noise ratio; see, e.g., [36,37]. However, calibration values for the DSCneed first to be estimated, before the DSI can be canceled [38]. The next processing step is the calibration of the antenna pattern, first in amplitude and second in phase using the MVPS. Finally, the clutter is removed via subtraction of the data.

We would like to mention that for the application of flex-DPCA, the knowledge of the receiver’s velocity is necessary. This can be achieved using an external velocity measuring device, e.g., an IMU. However, the receiver’s velocity can also be retrieved from the slope β of the clutter spectrum in the angle-Doppler domain, as the slope is directly related to the receiver’s velocity.

It is worth addressing the similarity between the approach presented here and the one suggested in [31,32]. Despite the fact that both algorithms have a common aim, namely to calibrate the receiving channels by exploiting the clutter returns, in order to suppress the clutter returns, there are significant differences between the approach from [31,32] and the approach described here. Specifically, the algorithm suggested in [31,32] employs a two-stage approach to minimize the clutter power level, by resorting to a least-squares approach to minimize the clutter power at the output of the DPCA subtraction stage, thus without considering the DPCA condition and the inherent nature of the clutter ridge in the angle-Doppler domain. A disadvantage of this approach is the susceptibility on targets with strong backscattering: strong backscattering targets might have a significant influence on the calibration coefficients, so that the target response might be significantly suppressed at the output of the DPCA stage. To prevent this, the size of the region in the range-Doppler map must be chosen carefully. However, as an advantage, the approach from [31,32] is computationally efficient, easily applicable, and considerably fast. Regarding internal clutter motion, which is indeed a problem in clutter suppression for GMTI, the approach is non-susceptible to internal clutter motion due to the minimization of the clutter power, thus being another advantage of this approach.

On the other hand, the approach proposed here obtains the channel calibration by first performing a coarse calibration using the direct signal [25] and second performing amplitude calibration. Finally, it resorts to a model-based approach, namely that the strongest eigenvector for any given Doppler-bin can be associated with the clutter component, following an approach also suggested in [39]. The underlying model implicitly associates Doppler frequency to AoA. This is exploited both for the amplitude calibration and for the phase calibration stages described above.

## 5. Application on Real Data

### 5.1. Measurement Campaign

A measurement campaign was conducted by Fraunhofer FHR. The trial was held in a rural area in Western Germany. As the receiving platform, a van pulling a trailer was used. The trailer carried a pedestal, which can be rotated in elevation and azimuth. It carried a linear array consisting of six discone antenna elements, where the four center elements were used as surveillance antennas and the two outer elements were terminated in order to achieve a uniform pattern of the surveillance elements. The array was mounted along-track.

**Figure 7:**
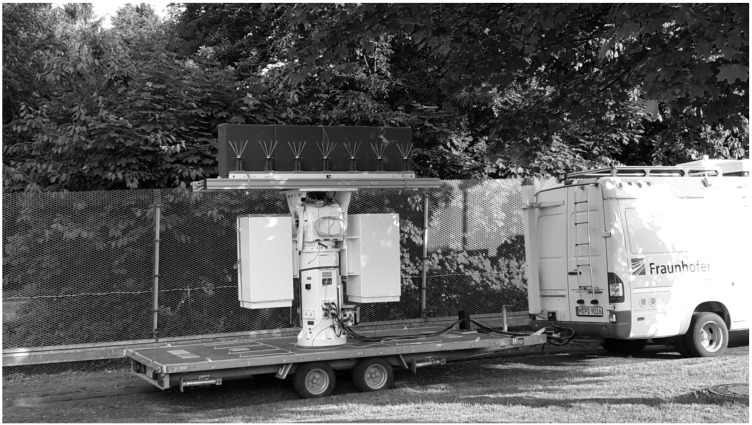
Picture of the bus with the trailer as the carrier platform. The uniform linear array is mounted on top of the white pedestal and steered along-track in the right-looking condition. The Radiation Absorbing Material (RAM) shielding the antennas to one side can be seen on top of the pedestal behind the antenna array.

To achieve a side-looking condition to one side only, Radiation Absorbing Material (RAM) was mounted along one side of the array. A picture of the experimental setup is reported in Figure 7.

As the receiving system, two Parasol units [40], developed by Fraunhofer FHR, were used. The Parasol system is a two channel receiving system with an effective sampled bandwidth of 32 MHz, built as a dual-superheterodyne receiver.

For this analysis, only the two central elements of the array were considered, thus downgrading the system to a two channel receiver.

To record the receiver’s position, velocity, and inertial movement, an IMU was mounted in the center of the array’s mounting separated from the antenna elements with the RAM, i.e., in the array’s back lobe.

**Figure 8:**
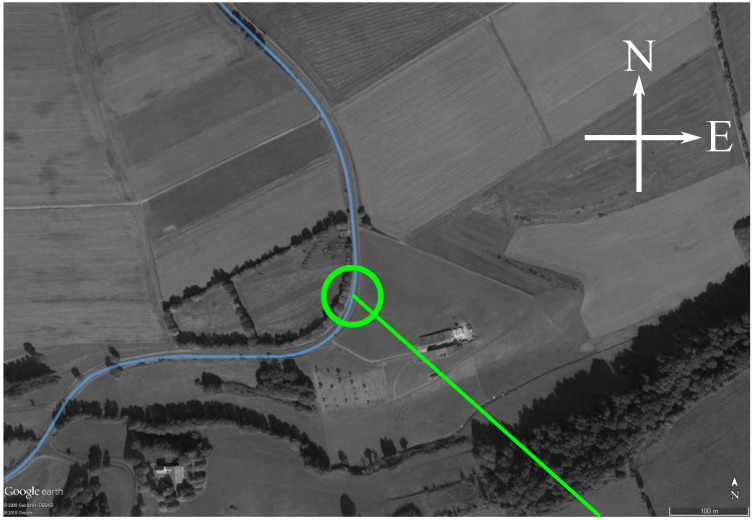
Picture of the trial site. The blue line indicates the trajectory of the receiver, while the green circle indicates its position at the time stamp of data evaluation. The green line indicates the baseline. Map data: ©Google, ©GeoBasis-DE/BKG 2009.

The exploited DVB-T transmitter “Tx Eifel” broadcasted at center frequencies $f_{C}=\left[674,690\right]$ MHz, where the DVB-T channel transmitted at $f_{C}=690$ MHz was exploited for the results presented here. Table 1 lists the relevant parameters of the exploited transmitter, the parameters during the acquisition, and the parameters of the signal processing. Figure 8 shows a map of the trial site. The receiver platform was moving from south to north along a street indicated in blue color. The position at the time stamp of the exploited data set is indicated with a green circle. The green line indicates the baseline connecting the positions of the receiver and transmitter. It is important to note that the antenna array was looking to the right side relative to the direction of movement; therefore, the direct signal at the exploited receiver position was received in the array’s main lobe, which is required for the first coarse calibration step, as described in Section 4.1.

### 5.2. Results

**Figure 9:**
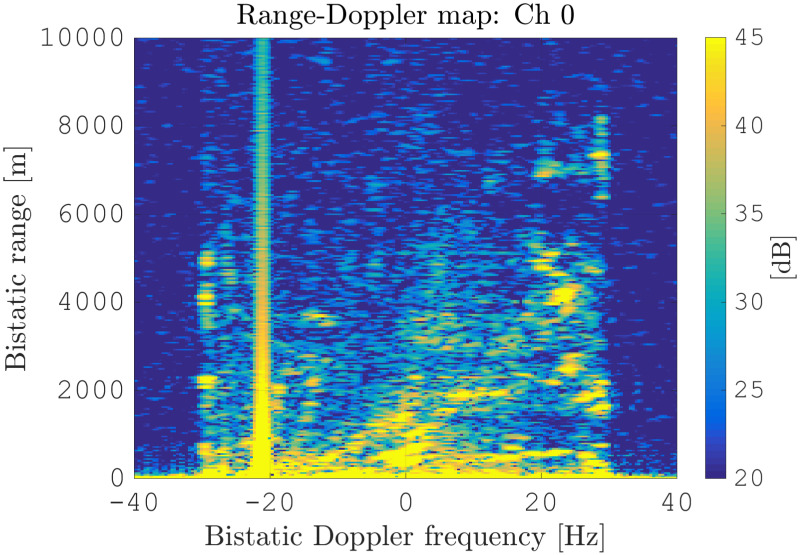
Initial range-Doppler map for the leading antenna.

An initial range-Doppler map of the LA is shown in Figure 9. One can see that the map is largely covered with clutter, thus preventing target detection. Especially at the near bistatic range up to 1000 m, the overall clutter power is high, which is due to the small grazing angle from the receiving array to the ground terrain. At Doppler $f_{D}=-22$ Hz, one can clearly see the interference from the direct signal.

As outlined, there are necessary calibration steps before clutter removal with DPCA can be done. For each single calibration step, the MVPS will be shown in order to analyze the clutter and confirm the theoretical analysis and simulation.

**Figure 10:**
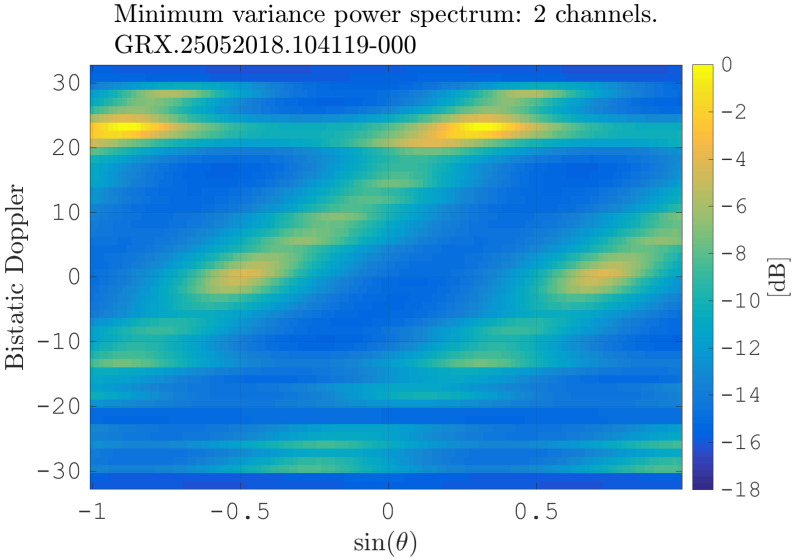
Minimum variance power spectrum of the data at the beginning of the processing chain.

Figure 10 shows the first estimation of the clutter spectrum in the angle-Doppler domain, i.e., before motion compensation or calibration. Before estimation of $P_{\mathrm{MV}}$, the covariance matrix R in (13) is diagonally loaded, in order to achieve convergence of the matrix inversion [41]. First of all, one can detect three clutter ridges in the angle-Doppler domain in Figure 10, which all have a linear slope, which aligns with the estimated slope $\beta =v_{\mathrm{Rx}}/d/\mathrm{PRF}$. It is difficult to tell the main lobe response, as due to unknown channel imbalances, no clutter spectra are centered at the center of the angle-Doppler domain at: $\bar{\theta }=0,f_{D}=0$, but instead deviate from the expected position. The ambiguous responses result from spatial undersampling as d/λ>0.5 ([23], p. 108). At approximately $f_{D}=-21$ Hz, there is no clutter response, as this Doppler refers to the Doppler of the DSI, which has been filtered using the Extensive Cancellation Algorithm by Carrier/Doppler (ECA-CD) filter [38], clearly showing the effect of direct signal cancellation. At $f_{D}\approx 25$ Hz, one can detect a strong response, which shows the presence of non-homogeneous clutter returns in the angle-Doppler domain.

**Figure 11:**
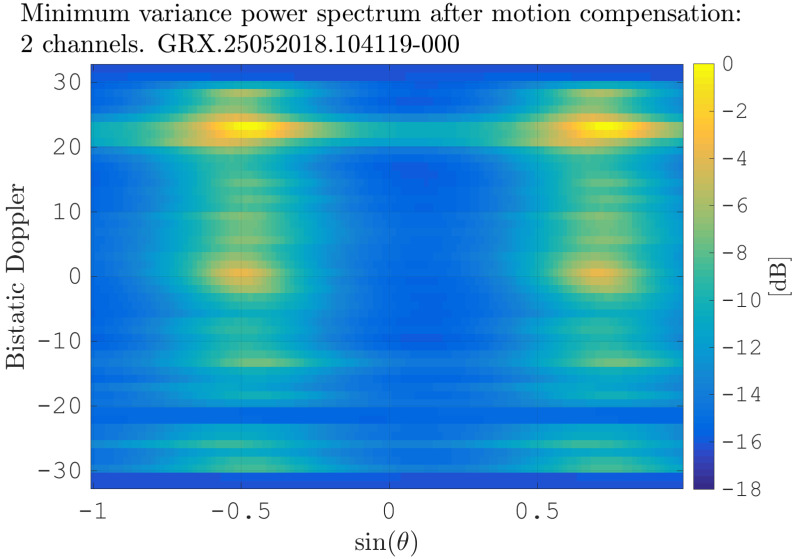
Minimum variance power spectrum after flex-DPCA application, i.e., after applying (11).

**Figure 12:**
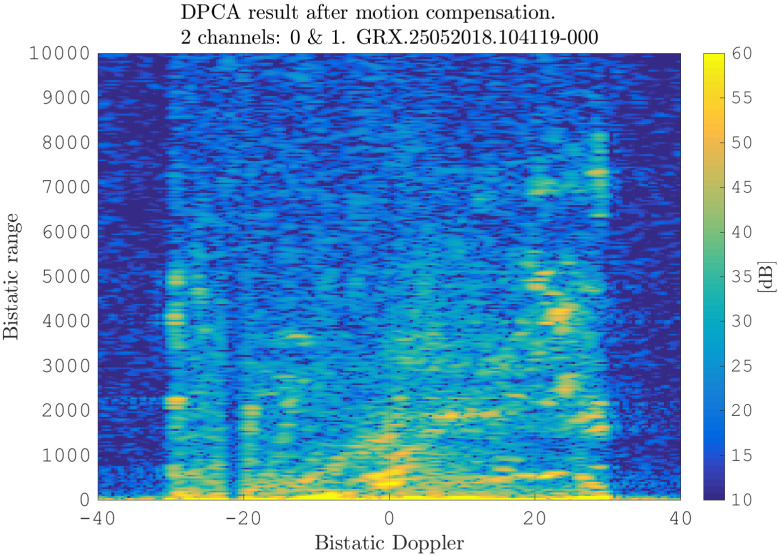
Initial range-Doppler map after DPCA (naive approach): only the motion compensation (11) and DPCA (12) were applied.

**Figure 13:**
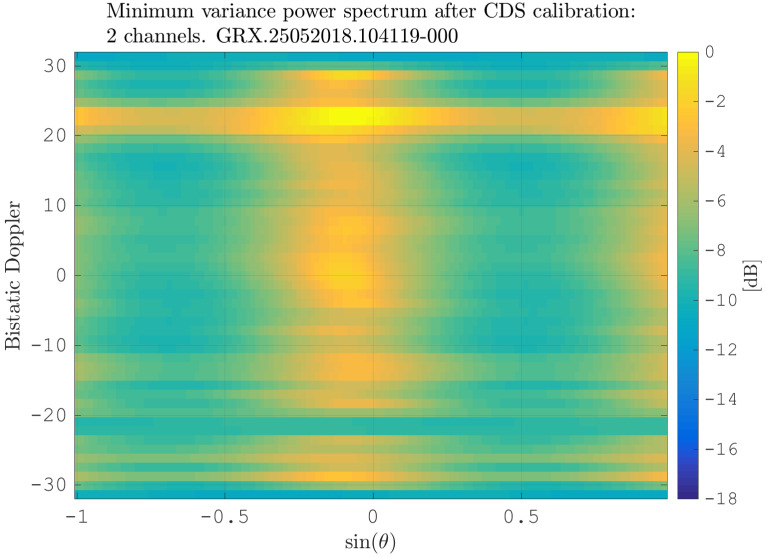
MV power spectrum after flex-DPCA and calibration using CDS.

**Figure 14:**
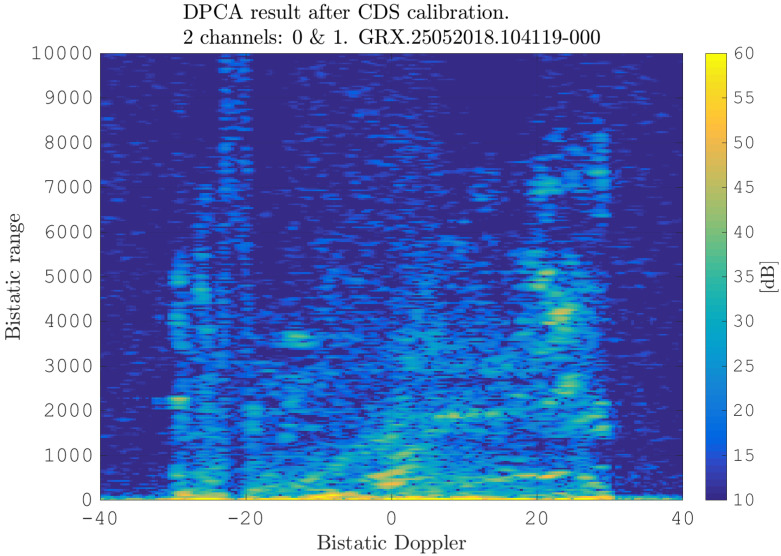
Range-Doppler map after calibration using CDS and clutter suppression using DPCA.

**Figure 15:**
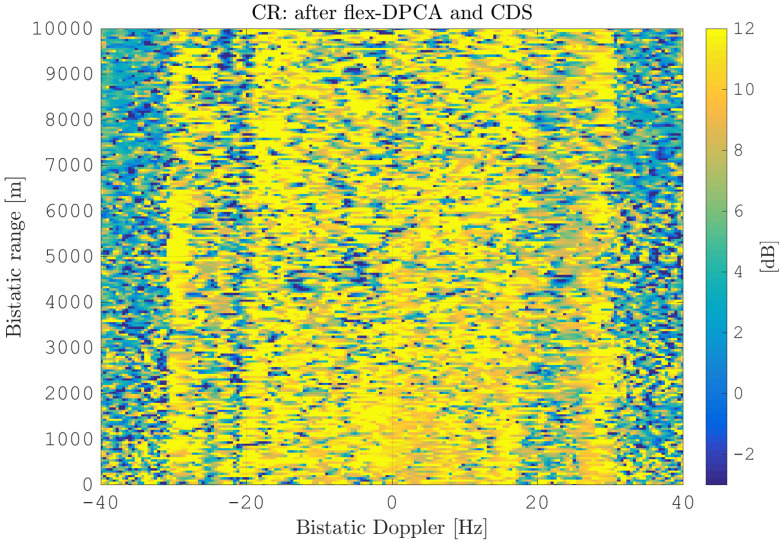
Cancellation ratio between range-Doppler maps after DPCA application: after flex-DPCA (Figure 12) and CDS (Figure 14).

**Figure 16:**
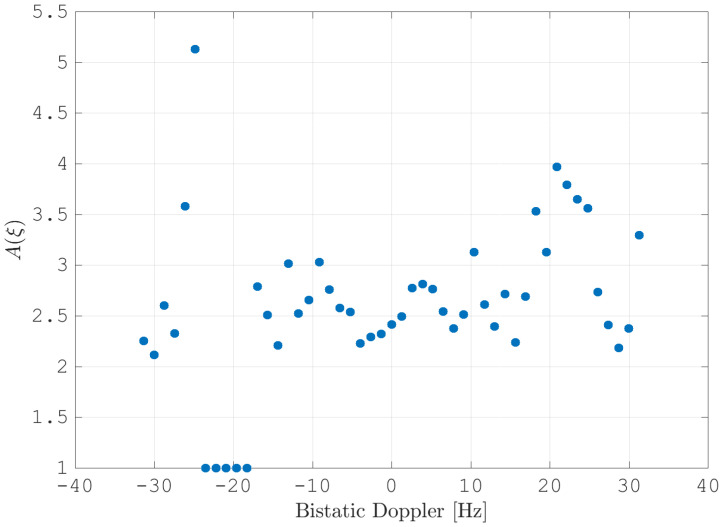
The estimated amplitude calibration values per Doppler-bin. The DSI at $f_{D}^{\left(\mathrm{Tx}\right)}=-11$ Hz was suppressed; therefore, the Doppler-bin at $f_{D}^{\left(\mathrm{Tx}\right)}$ and Doppler-bins next to $f_{D}^{\left(\mathrm{Tx}\right)}$ were excluded from the estimation and calibration.

**Figure 17:**
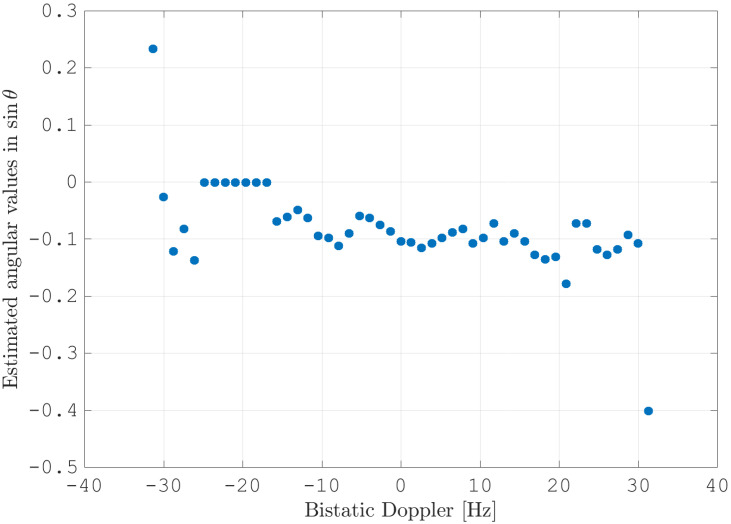
The estimated calibration values for the phase per Doppler-bin. The DSI at $f_{D}^{\left(\mathrm{Tx}\right)}=-11$ Hz was suppressed; therefore, the Doppler-bin at $f_{D}^{\left(\mathrm{Tx}\right)}$ and Doppler-bins next to $f_{D}^{\left(\mathrm{Tx}\right)}$ were excluded from the estimation and calibration.

After estimating the time delay values with (10) and applying (11), the MVPS can be estimated again, which is shown in Figure 11. Clearly, in accordance with the analysis of the outcome of the signal analysis and with Figure 2, the clutter spectrum is vertical and parallel to the bistatic Doppler axis. However, grating lobes in the clutter spectra still appear. Furthermore, the variance of the angular deviations is unequal zero; that means the centroid of the angular values in each Doppler-bin deviates from the mean value, which is due to irregularities in the antenna pattern. Although applying DPCA at this stage would not lead to a clutter suppression, we report here the outcome of DPCA in Figure 12 for comparison reasons. Comparing Figure 12 with Figure 9, it becomes clear that the naive approach of simple motion compensation without calibration does not remove the clutter. The first calibration step is the coarse calibration on the direct signal interference. The calibration values according to (15) were estimated before the direct signal was filtered with the ECA-CD. Afterwards, the $Z^{\left(\mathrm{TA}\right)}$ was calibrated according to (16). The estimation of $P_{\mathrm{MV}}$ after CDS calibration is reported in Figure 13. In comparison to Figure 11, one can clearly see that the clutter spectra in the angle-Doppler domain are projected on the angular axis, so that one spectrum is closer to the zero-angle axis at $\bar{\theta }=0$, and it can be assumed that the spectrum is resulting from main lobe reception. This calibration provided an equalization in phase and amplitude of the antenna patterns for the AoA of the direct signal. To suppress the clutter, the calibrated data ${\hat{Z}}^{\left(\mathrm{TA}\right)}$ were plugged into (8). The outcome is reported in Figure 14, and to support the comparison, the cancellation ratio between Figure 12 and Figure 14 is reported in Figure 15. Despite this simple approach, a clutter subtraction is achieved by up to 12 dB, where the suppression is uniform across the range Doppler map, due to the equalization using one calibration factor only. However, the range-Doppler map is still largely covered by clutter not being suppressed; especially, it is covered by heterogeneous clutter, which is challenging to remove by algorithms that rely on filter estimation based on clutter statistics, e.g., by STAP. Therefore, we applied the antenna pattern calibration in amplitude and phase using the MVPS, in order to be independent from the clutter statistics. Following (17), we calibrated first the amplitude, then the phase. The estimated values are shown in Figure 16 and Figure 17. In Figure 17, one can clearly see a sinusoidal trend of the phase calibration values, which justifies also the parameters of the simulation.

**Figure 18:**
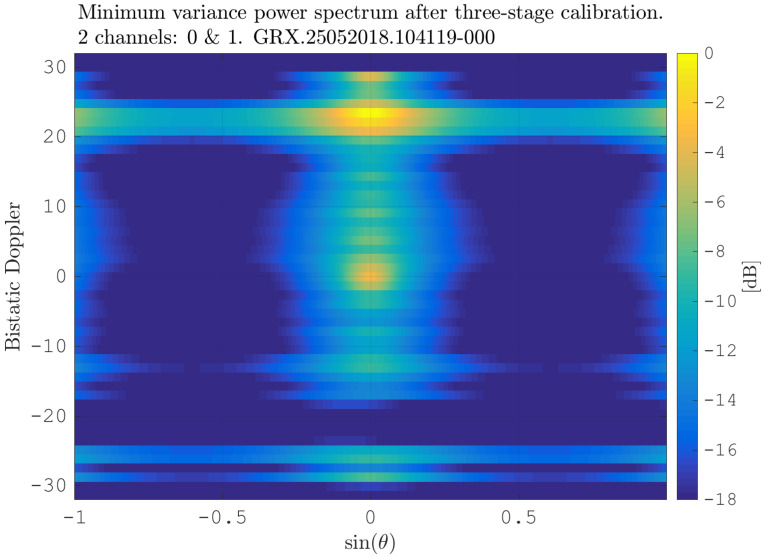
MV power spectrum after calibrations using CDS and MVPS.

**Figure 19:**
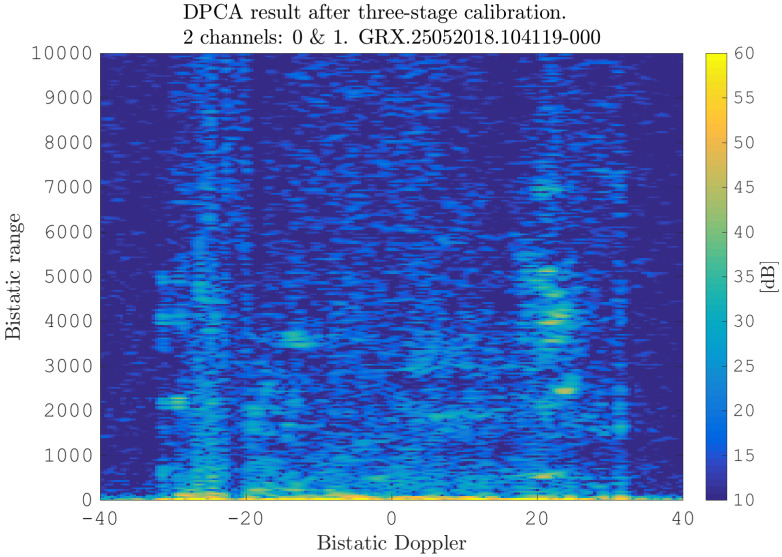
Range-Doppler map after calibrations using CDS and MVPS and clutter suppression using DPCA.

**Figure 20:**
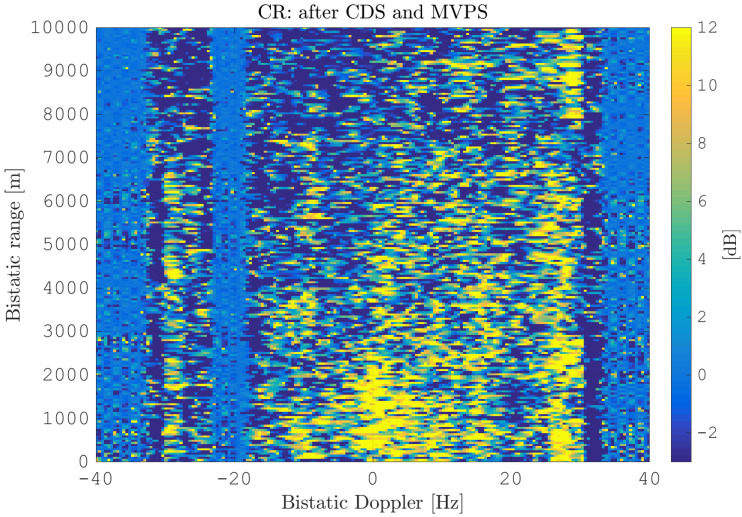
Cancellation ratio between range-Doppler maps after DPCA application: between calibration with CDS (Figure 14) and calibration with MVPS (Figure 19).

After this calibration, the MVPS is clearly centered at $\bar{\theta }=0,\;\forall f_{D}$, visible in Figure 18. This allows effectively suppressing the clutter with DPCA, presented in Figure 19. Comparing Figure 19 to Figure 14 clearly shows the improvement of further clutter suppression. Especially, the heterogeneous clutter is widely suppressed, e.g., at bistatic Doppler $f_{D}=\left[-20,\ldots ,0\right]$ Hz and bistatic range $r_{B}=\left[200,1500\right]$ m or $f_{D}=\left[-5,\ldots 5\right]$ Hz and $r_{B}=\left[200,2000\right]$ m. This can be also observed at other regions in the range-Doppler map; see the plot of the cancellation ratio reported in Figure 20. This figure shows the incremental cancellation obtained using the new calibration technique, over the simple CDS calibration. Clearly, one can see that the heterogeneous clutter is further largely suppressed; see especially the region at the bistatic Doppler from −15 Hz up to 20 Hz and the bistatic range up to 2000 m. In the region of the maximum clutter Doppler frequency around 30 Hz for all bistatic ranges as well, the clutter is significantly reduced.

This result proves the feasibility and effectiveness of the presented approach in terms of clutter suppression, which is expected to allow the detection of slow moving targets.

## 6. Conclusions

In this paper, we considered the problem of clutter subtraction for a passive radar on a moving platform with the goal to detect slow moving targets hidden in the clutter. Specifically, we focused on the limitations in clutter subtraction arising from non-calibrated antenna patterns. To overcome this limitation, we proposed an effective processing scheme for angle-dependent antenna pattern calibration. The processing scheme involved an initial motion compensation step, called flex-DPCA, which we analyzed by exploiting the MVPS in a simulation and justified mathematically. We made the observation that in an ideal environment, the MVPS is projected on the temporal axis in the clutter-angle domain. Based on this observation, we introduced a calibration processing scheme, which first exploits the direct signal interference for a coarse pattern equalization in amplitude and phase, and then, the MVPS is exploited to calibrate the amplitude and angle of the antenna pattern on an angle-dependent basis. The effectiveness of the proposed solution was investigated by means of application to an experimental data set. The results clearly demonstrate the enhancement of our approach in terms of clutter suppression, especially against heterogeneous clutter, which is challenging to suppress with data-adaptive methods. Future research will include the exploitation of multiple receiving channels to further enhance clutter suppression.

## Figures and Tables

**Table 1:** List of parameters of the analyzed DVB-T signal, as well as the parameters of the measurement campaign and signal processing.

Symbol	Description	Value
**DVB-T signal parameters**		
	DVB-T standard mode	8k
$f_{C}$	Carrier frequency	674, 690 (MHz)
$T_{S}$	OFDM symbol duration	1120 (μs)
$T_{U}$	Duration of the useful symbol part	896 (μs)
$T_{G}$	Duration of the guard interval	224 (μs)
**Measurement campaign and signal processing parameters**		
$v_{\mathrm{Rx}}$	Platform velocity	≈12.6 m/s
*d*	Employed antenna spacing	0.36 m
CPI	Coherent Processing Interval	$709\cdot T_{S}$

## Data Availability

Data sharing is not applicable to this article.
